# Socio‐economic inequality in health service utilisation: Does accounting for seasonality in health‐seeking behaviour matter?

**DOI:** 10.1002/hec.3925

**Published:** 2019-07-02

**Authors:** John E. Ataguba

**Affiliations:** ^1^ Health Economics Unit, School of Public Health and Family Medicine, Health Sciences Faculty University of Cape Town Cape Town South Africa

**Keywords:** concentration index, seasonal variation, seasonality index, socio‐economic inequality

## Abstract

Seasonal variation exists in disease incidence. The variation could occur across the different regions in a country. This paper argues that using national household data that are not adjusted for seasonal and regional variations in disease incidence may not be directly suitable for assessing socio‐economic inequality in annual outpatient service utilisation, including for cross‐country comparison. In fact, annual health service utilisation may be understated or overstated depending on the period of data collection. This may lead to miss‐estimation of socio‐economic inequality in health service utilisation depending, among other things, on how health service utilisation, across geographical areas, varies by socio‐economic status. Using a nationally representative dataset from South Africa, the paper applies a seasonality index that is constructed from the District Health Information System, an administrative dataset, to annualise public outpatient health service visits. Using the concentration index, socio‐economic inequality in health service visits, after accounting for seasonal variations, was compared with that when seasonal variations are ignored. It was found that, in some cases, socio‐economic inequality in outpatient health service visits depends on the socio‐economic distribution of the seasonality index. This may justify the need to account for seasonal and geographical variations.

## 1 INTRODUCTION

Disease incidence may follow a seasonal pattern (Briet, 2002; Sauerborn, Nougtara, Hien, & Diesfeld, 1996). This is not limited to infectious diseases where such seasonal variation is well documented (Altizer et al., 2006; Grassly & Fraser, 2006). Seasonal variation may also exist for other conditions such as the incidence of mental illness (Tyrer et al., 2016) and emergency general surgery (Zangbar et al., 2016). In many parts of sub‐Saharan Africa, for instance, malaria accounts for a considerable burden of disease, and its incidence is seasonal (Cairns et al., 2012; Mabaso, Craig, Vounatsou, & Smith, 2005; Zhou, Minakawa, Githeko, & Yan, 2004). In the context of socio‐economic inequalities in health, seasonality indices have been suggested to annualise health service utilisation (McIntyre & Ataguba, 2011), but there is a dearth of studies that show the implications of ignoring seasonality. Theoretically, seasonality indices are in part necessary as household data collected for assessing inequality and inequity in health service utilisation are mainly cross‐sectional and often use a fixed recall period. These surveys are also conducted within a particular time of the year, corresponding to different seasons in different regions or countries. Four or two weeks are the most common recall periods for outpatient service utilisation, whereas inpatient admissions use a 6‐ or 12‐month recall period. The Demographic and Health Surveys are examples of household survey data that collect information on health service utilisation (see McIntyre & Ataguba, 2011, for a discussion on some issues relating to the use of different datasets for health inequality/equity analysis).

Traditionally, reported outpatient service utilisation data are annualised by multiplying them by 12 if the recall period was the last 1 month (O'Donnell, Van Doorslaer, Wagstaff, & Lindelow, 2008). However, if data were collected during a season of low (or high) disease incidence and they are annualised using a uniform scalar, all things being equal, annual disease burden and annual health service utilisation may be underestimated (or overestimated). The underestimation or overestimation of health service utilisation may affect socio‐economic groups differently depending on, for example, the relationship between disease incidence and household location. In fact, there is a correlation between the spatial distribution of people and the extent to which they are exposed to different health conditions (Blanchard, Bernstein, Wajda, & Rawsthorne, 2001), and this could lead to variations in health service utilisation patterns. Because these variations due to differential location (and disease incidence) may affect different socio‐economic groups in different ways, it is essential to consider that in assessing socio‐economic inequality in health service utilisation. Also, cross‐country comparisons of health inequalities are difficult to achieve without accounting for seasonal and regional variations in health service utilisation within countries. Thus, this paper constructed seasonality indices using information from the District Health Information System. The seasonality indices are used to assess the sensitivity of socio‐economic inequalities in public outpatient health service visits to seasonal and regional variations in disease incidence using household data. Using data from South Africa, this paper found that, after accounting for seasonal variations, socio‐economic inequality in outpatient health service visits depends on the socio‐economic distribution of the seasonality index only in a few cases. Although this is the case for South Africa, the significance may be pronounced in other country applications, thus justifying the need to account for seasonal and geographical variations.

## 2 METHODS

The empirical application is based on data from South Africa, a country with both public and private health sectors. Public health services are delivered through public clinics, community health centres, and hospitals (ranging from district to national central hospitals). This paper uses nationally representative South African Consortium for Benefit Incidence Analysis (SACBIA) survey 1The SACBIA survey was a collaborative initiative by the Health Economics Unit, University of Cape Town; Centre for Health Policy, University of the Witwatersrand; the National Department of Health; and the London School of Hygiene and Tropical Medicine. SACBIA was funded by the South African National Department of Health and the European Union, and data were collected by the Community Agency for Social Enquiry. data. Among other things, the SACBIA dataset contained reliable data on health service utilisation. Details on the study area and the data structure including the sampling procedure have been described elsewhere (Ataguba & McIntyre, 2013). Data were collected over 3 months between April and July 2008 in all nine South African provinces. A total of five households were selected from each of the 960 enumeration areas identified. The total sample size was 4,800 households (approximately 22,000 individuals). Health service utilisation data include the use of inpatient and outpatient services at both public and private facilities. The recall period for inpatient service utilisation was 12 months, whereas 1 month was used for outpatient services. Thus, only outpatient visits are used in this paper as no further annualisation was required for inpatient services.

Two forms of annualisation were performed, and the results of socio‐economic inequality in public outpatient health service visits were compared between them. The first, also called uniformly annualised visits, was done by multiplying public outpatient visits by 12 (this assumes the same utilisation pattern throughout the year; O'Donnell et al., 2008). 2It is important to note that relative socio‐economic inequality in health service utilisation will not change when utilisation is multiplied by a uniform scalar (O'Donnell et al., 2008). The other (i.e., seasonally annualised visits) involved annualising public outpatient visits using a seasonality index generated on the basis of aggregate public outpatient visits in each district recorded in the District Health Information System (DHIS), a form of the census on public health service utilisation. Because the DHIS contains data on public facility utilisation, only public outpatient visits disaggregated by health provider level (clinics, district hospitals, etc.) were considered in this paper. The disaggregation does not contain information on specific diseases.

The SACBIA dataset was representative at the province and not the district level; thus, province‐specific seasonality indices were used. The seasonality index for month *j* (i.e., the month that the survey was conducted in each province), for visits to facility type *k* in province *l* (i.e., ${\mathrm{SI}}_{\mathrm{jk}}^{l}$), is defined as follows:

(1) $${\mathrm{SI}}_{\mathrm{jk}}^{l}=\frac{\sum_{i=1}^{12}U_{\mathrm{ik}}^{l}}{U_{\mathrm{jk}}^{l}},$$

where $U_{\mathrm{ik}}^{l}$ is the total visits to a specified facility type *k* in month *i* in province *l*, and $U_{\mathrm{jk}}^{l}>0$ is the total visits to facility type *k* in month *j* in province *l*. For example, if for visits to a given facility type or level (e.g., public clinics), the seasonality index $\left({\mathrm{SI}}_{\mathrm{jk}}^{l}\right)$ computed for a given month ( *j*) in a specific province (*l*) was estimated to be less than (greater than) 12, it means that uniformly annualising health service utilisation using month *j*'s utilisation will overstate (understate) annual health service utilisation as health service utilisation in that month is greater (lesser) compared with the overall monthly average.

Concentration indices were used to assess socio‐economic inequality in public outpatient visits (after uniformly or seasonally adjusting the visits) and socio‐economic inequality in the seasonality indices. 3Although the primary interest of this paper is to assess the implications of seasonal variations in health service utilisation on socio‐economic inequality, socio‐economic inequality in the seasonality indices was used in this paper to, among other things, confirm the relationship developed later in Equation (3). A concentration index is obtained from the concentration curve that plots the cumulative percentage of health service utilisation (or seasonality index) against the cumulative percentage of the population, ranked by a measure of socio‐economic status (Erreygers, 2009; Kakwani, Wagstaff, & van Doorslaer, 1997; Wagstaff, 2005; Wagstaff, Paci, & van Doorslaer, 1991). Socio‐economic inequality in the health service utilisation or seasonality indices shows the extent to which health service utilisation or the seasonality indices are concentrated among different socio‐economic groups.

The concentration index (*C*_*H*_) was computed as presented in Araar and Duclos (2009b):

(2) $$C_{H}=1-\left({\hat{\xi }}_{H}/{\hat{\mu }}_{H}\right),$$

where ${\hat{\mu }}_{H}$ is the weighted average of the health service utilisation or seasonality index, ${\hat{\xi }}_{H}=\sum_{i=1}^{n}\left(\left(V_{i}^{2}-V_{i+1}^{2}\right)/V_{1}^{2}\right)h_{i}$, $V_{i}=\sum_{m=i}^{n}w_{m}$. The vector **w** = [*w*_1_, *w*_2_,  … , *w*_*n*_] is the sampling weights and the vector of per adult equivalised household consumption (i.e., the proxy for income or ranking variable) **X** = [*x*_1_, *x*_2_,  … , *x*_*n* − 1_, *x*_*n*_].

In this paper, these indices (*C*_*H*_) are computed using the DASP routine (Araar & Duclos, 2009a) in Stata 15 (StataCorp, 2017). Briefly, the concentration index shows the extent to which health service utilisation (or seasonality index) occurs more among the richer or poorer segment of the population—the values range from −1 to +1. A positive (negative) concentration index means that richer (poorer) households or individuals use more health services compared with their poorer (richer) counterparts. Stated differently, a positive concentration index implies a prorich distribution, whereas a negative index implies a propoor distribution. Relatedly, although not presented in this paper, horizontal inequity (i.e., a case where people with equal need do not receive the same care or treatment; Wagstaff, Van Doorslaer, & Paci, 1991) can also be computed using concentration indices, as the difference between the concentration index for health service utilisation and that for need (Jui‐fen et al., 2007; Phiri & Ataguba, 2014).

Generally, the difference in magnitude (and sometimes the sign) between the concentration index of the seasonally annualised visits (*CI*_*SA*_) and the concentration index of the uniformly annualised visits (*CI*_*UA*_) is determined by the sign of the concentration index of the seasonality index (*CI*_*SI*_).

(3) $${\mathrm{CI}}_{\mathrm{SA}}\left\{\begin{array}{c} >{\mathrm{CI}}_{\mathrm{UA}}\\ <{\mathrm{CI}}_{\mathrm{UA}}\\ ={\mathrm{CI}}_{\mathrm{UA}} \end{array}\right.\;\begin{array}{c} \text{if}\\ \text{if}\\ \text{if} \end{array}\;\begin{array}{c} {\mathrm{CI}}_{\mathrm{SI}}>0\\ {\mathrm{CI}}_{\mathrm{SI}}<0\\ {\mathrm{CI}}_{\mathrm{SI}}=0. \end{array}$$

Briefly, as shown in Table 1, when *CI*_*SI*_ < 0, and seasonal variations have not been accounted for, estimated inequality (*CI*_*UA*_) will be biased in favour of richer individuals or households. So, accounting for seasonal variations in this case will “increase” the concentration among poorer individuals or households. The reverse is the case when *CI*_*SI*_ > 0.

**Table 1:** The differences between socio‐economic inequality in uniformly and seasonally annualised visits

Scenario	Description
*CI*_*SA*_ > *CI*_*UA*_	In a simple visual display, this occurs when the concentration curve for the seasonality index lies below the line of equality, irrespective of a propoor or prorich distribution of the uniformly annualised visits. For example, if the distribution of the uniformly annualise visits is propoor (i.e., *CI*_*UA*_ < 0), then it must be the case that the distribution of the underlying seasonality index was prorich (i.e., *CI*_*SI*_ > 0) for *CI*_*UA*_ < *CI*_*SA*_. This is also the case when *CI*_*UA*_ > 0.
*CI*_*SA*_ < *CI*_*UA*_	Similarly, this is the case where the concentration curve for the seasonality index lies above the line of equality, regardless of the propoorness or prorichness of the uniformly annualised visits. For example, if *CI*_*UA*_ > 0, such that uniformly annualised visits have a prorich distribution, then *CI*_*UA*_ > *CI*_*SA*_ is a result of a negative value for *CI*_*SI*_. The same applies to a case where *CI*_*UA*_ < 0.
*CI*_*SA*_ = *CI*_*UA*_	This means that there is no difference between the two distributions (*UA* and *SA*) as the distribution of seasonally annualised visits is the same as that for uniformly annualised visits. Therefore, *CI*_*SI*_ = 0. Theoretically, this means that the concentration curve of the seasonality index coincides with the line of equality. However, this may not always be the case as curves may cross with a zero net effect.

*Note*. The distribution of the seasonality index determines the difference between *CI*_*UA*_ and *CI*_*SA*_.

As the difference between these indices (*CI*_*UA*_ and *CI*_*SA*_) is computed on the basis of underlying concentration curves, a statistical test for dominance was performed using the intersection and union approach as detailed in O'Donnell et al. (2008). 4The “dominance” command written by Owen O'Donnell was used to perform statistical dominance tests (http://siteresources.worldbank.org/INTPAH/Resources/Publications/459843-1195594469249/dominance.ado). This is supplemented with a second‐order stochastic dominance test (Araar & Duclos, 2009a). As explained elsewhere (Ataguba & McIntyre, 2013), because income is not reliable in the context of South Africa, the household living standard or income was proxied by per adult equivalised household consumption. 5The adult equivalent scale (*AE*) used is as follows: *AE* = (*A*+*αC*)^*θ*^, where *A* is the number of adults in the household, *C* is the number of children, *α* is the cost of children (a measure of the weight accorded to children relative to adults; Banks & Johnson, 1994), and *θ* represents economies of scale. As recommended in Deaton and Zaidi (2002), we set *α* = .5 and *θ* = .75. In fact, the results were identical for when we used the per capita household consumption.


## 3 RESULTS AND DISCUSSION

The average values of the seasonality indices increase with household income deciles (Table 2). The average seasonality indices for visits to public health services in South Africa are generally lower in deciles 1–5 compared with the national averages. The results in Table 2 are confirmed in Table 3, where the concentration indices are positive—socio‐economic inequality in the seasonality indices shows a prorich distribution for visits to all public health services (i.e., *CI*_*SI*_ > 0).

**Table 2:** Average seasonality index by socio‐economic deciles

Decile	District hospital	Regional hospital	Provincial tertiary hospital	National central hospital	Clinics and community health centres
D1 (poorest)	11.33	10.19	11.12	12.30	11.88
D2	11.34	10.22	11.15	12.29	11.89
D3	11.36	10.25	11.20	12.32	11.90
D4	11.35	10.24	11.14	12.26	11.88
D5	11.35	10.25	11.15	12.25	11.88
D6	11.37	10.28	11.19	12.25	11.89
D7	11.37	10.28	11.22	12.30	11.91
D8	11.37	10.30	11.23	12.27	11.91
D9	11.40	10.33	11.37	12.42	11.97
D10 (richest)	11.44	10.38	11.52	12.56	12.04
National	11.37	10.27	11.23	12.32	11.91

**Table 3:** Socio‐economic inequality in the seasonality index

Public health facility	Concentration index
Clinics and community health centres	0.0018* (0.0004)
District hospital	0.0014* (0.0002)
Regional hospital	0.0028* (0.0004)
Provincial tertiary hospital	0.0050* (0.0010)
National central hospital	0.0026* (0.0008)

*Note*. The total sample size was 21,158 individuals; standard errors in parenthesis.

* Statistically significant at 1% level.

On the basis of the socio‐economic distribution of the seasonality indices shown in Table 3 (*CI*_*SI*_ > 0), and as discussed in Equation (3), it is expected that *CI*_*SA*_ > *CI*_*UA*_. In fact, this is seen in the results in Table 4. For example, the use of public clinics and community health centres became less propoor after accounting for seasonal variations in public outpatient visits. The same pattern is observed for the visits to public district hospitals and provincial tertiary hospitals. Similarly, the use of regional and national central hospital outpatient services became more prorich after applying the seasonality index. Some of these differences (*CI*_*UA*_ − *CI*_*SA*_) in Table 4, although relatively small in magnitude, are statistically significant. For example, the decreased propoorness of visits to district hospitals was confirmed to be statistically significant at the 5% level, whereas the increased prorichness of visits to regional hospitals was confirmed to be statistically significant at the 1% level. Using statistical dominance tests, only regional hospital outpatient services show a statistically significant difference between the uniformly and seasonally annualised visits. Additionally, there was stochastic dominance for visits to district hospitals. In some cases, the concentration curves cross each other, or there is no clear dominance between curves.

**Table 4:** Socio‐economic inequality in public health service utilisation using uniform and seasonally annualised visits

Public health facility	*CI*_*SA*_ (1)	*CI*_*UA*_ (2)	Difference (3) = (2) − (1)	Dominance	
				IU	SSD
Clinics and community health centres	−0.1337*** (0.0251)	−0.1342*** (0.0250)	−0.0005 (0.0007)	CX	CX
District hospitals	−0.2245*** (0.0425)	−0.2259*** (0.0425)	−0.0015** (0.0007)	ND	2D1
Regional hospitals	0.0143 (0.0661)	0.0103 (0.0660)	−0.0040*** (0.0013)	2D1	2D1
Provincial tertiary hospitals	−0.0012 (0.0730)	−0.0073 (0.0720)	−0.0060* (0.0032)	ND	CX
National central hospitals	0.3578*** (0.1000)	0.3543*** (0.0977)	−0.0036 (0.0032)	ND	CX

*Note*. The total sample size was 21,158 individuals; standard errors in parenthesis; the statistical significance of the difference between *CI*_*SA*_ and *CI*_*UA*_ was assessed using analytic standard errors.

Abbreviations: 2D1, the concentration curve of uniform annualised visits dominates the concentration curve of seasonally annualised visits; CX, curves cross; IU, intersection and union dominance; ND, nondominance; SSD, second‐order stochastic dominance using the DASP routine (Araar & Duclos, 2009a).

* Statistically significant at 10% level.

** Statistically significant at 5% level.

*** Statistically significant at 1% level.

Overall, using data collected between April and July 2008, although some of the results are not significant, they indicate the importance of accounting for seasonal and regional variations in disease patterns within a country. Here, the disease pattern was proxied by reported utilisation obtained from the comprehensive DHIS database. In this case, there is a statistically significant and systematic variation in the distribution of the seasonality indices in favour of the rich. If this variation is not accounted for within a country, socio‐economic inequalities may be overstated in favour of the poor, when an annual generalisation is intended. It is important to note that the prorich pattern obtained in Table 3 may not always be the case as this was specific to the period when the SACBIA data were collected. Thus, it cannot be generalised that outpatient health service utilisation (even in South Africa) will be biased in favour of the poor when anualisation is based on a uniform scalar. In fact, as shown in Equation (3), it is possible for *CI*_*SI*_ < 0, such that an already propoor pattern (*CI*_*UA*_) could become even more propoor (*CI*_*SA*_). Thus, it is important to first understand the socio‐economic distribution of the seasonality index within each context to conclude on the direction of the relationship between socio‐economic position and annual utilisation of health services.

## 4 CONCLUSION

Theoretically, as shown in Equation (3), accounting for seasonality impacts on the assessment of socio‐economic inequality. Using data from South Africa, this paper provides evidence that accounting for seasonal patterns in health service utilisation across provinces in South Africa may significantly impact on the socio‐economic distribution of annualised outpatient health service utilisation using concentration indices. Here, the seasonality indices are concentrated among the rich, such that *CI*_*SA*_ > *CI*_*UA*_. Overall, there is evidence, although not in all cases, suggesting that accounting for seasonal variations in disease incidence or utilisation patterns is necessary when annualising health service utilisation data for assessing socio‐economic inequalities in health utilisation. If seasonality is to be ignored, results must be interpreted accordingly to reflect the period that the data were collected and may not be comparable between countries.

## ETHICAL STATEMENT

The original study from where data are obtained had received ethical approval from the University of Cape Town.
